# Using “Own-Point-of-View” Video and Stimulated Recall to Reveal Physician Thought Processes: A Methodology Paper

**DOI:** 10.5811/westjem.54032

**Published:** 2026-07-16

**Authors:** Milisa Manojlovich, Caitlin Cassady, Sarah J. Parker, Ellie Davis, Charlotte Ahr, David Ryamukuru, Anna Wang, Kalyan Pasupathy, Hardeep Singh, Prashant Mahajan

**Affiliations:** * University of Michigan School of Nursing, Ann Arbor, Michigan; † University of Michigan, Department of Emergency Medicine, Ann Arbor, Michigan; ‡ University of Illinois at Chicago, Chicago, Illinois; § Baylor College of Medicine, Michael E. DeBakey Veterans Affairs Medical Center, Center for Innovations in Quality, Effectiveness and Safety, Houston, Texas

## Abstract

**Introduction:**

Video methods used in medicine have been described as the “gold standard” because they show events as they occurred and with a level of detail unattainable when events are reconstructed using other methods. In stimulated recall, video or pictures during interviews prompt discussion about participants’ thoughts or feelings when images were captured. Coupled with video, stimulated recall can deepen researchers’ understanding of a participant’s thinking at the time. However, conducting video-based stimulated recall interviews with participants is poorly described in the literature because of word count limits in today’s journals. In this paper we describe how we used video with stimulated recall interviews to learn how the diagnostic process evolves for emergency physicians.

**Methods:**

We used the theory of distributed cognition to describe how information processing develops over time and occurs across physicians, the technologies used, and social organization. We conducted a qualitative study, collecting data in pediatric and adult emergency departments (ED) of an academic Level I trauma center. Participants wore head-mounted video cameras for two hours while providing care to ED patients and revealed their thinking throughout the diagnostic process in subsequent stimulated recall interviews. We reviewed video recordings to identify situations related to the diagnostic process or to one of the concepts in distributed cognition. We identified short video clips from each session to display during interviews and prepared accompanying questions. Stimulated-recall interviews were conducted using video conferencing technology, audio recorded, transcribed, and verified for accuracy. We used content analysis to analyze results.

**Results:**

Eleven attending physicians from February 2022–May 2023 (five from the pediatric ED and six from the adult ED) interacted with 52 patients over a total of 24.4 hours on video. We obtained patients’ permission to film encounters before a participating physician provided care, wearing the head-mounted camera. We conducted individual stimulated recall interviews (mean 53 minutes, range 33–63 minutes) with all 11 physicians, which uncovered perspectives beyond what was revealed on film. For example, the complexity of cognition resides in patients as well as physicians. Thus, physicians must exert great effort to elicit the patient story and make a likely diagnosis in the chaotic, noisy environment of the ED.

**Conclusion:**

Using innovative methods to uncover emergency physicians’ cognitive processes has the potential to advance our understanding in other settings. This methodology brings awareness to how one’s thoughts can become visible and, thus, amenable to reflection and change.

## INTRODUCTION

Video-based methods have been used in medicine for over 50 years.^1^ Video has been described as the “gold standard” because it shows events as they occurred and with a level of detail unattainable when events must be reconstructed using other methods.^2^ Video can be re-viewed multiple times to facilitate viewer interpretation. Stimulated recall is a procedure in which video or pictures are used during interviews to prompt discussion about participants’ thoughts or feelings at the time when the images were captured^3^ since the images themselves do not display participants’ thought processes.^4^ When coupled with video, stimulated recall can be used to deepen researchers’ understanding of a participant’s thinking at the time.^3^ Thus, the pairing of video data and simulated recall are especially suited to learn more about cognitive processes and decision-making in medicine.

Video-stimulated recall can be done in a variety of ways.^5^ The decision should rest on the type of data the researcher aims to gather, whether that be recall, re-living, or reflection, all of which can be produced by stimulated recall interviews.^5^,^6^ Data quality can be enhanced when researchers design their studies to focus on the kind of data they want.^6^ For example, the usual video portion of the methodology involves focusing the camera on the participant and capturing interactions between the participant and others. An adaptation of the method is to have participants wear head-mounted cameras so that the camera captures what the participant is looking at (“own point-of-view”), prioritizing the participant’s choice of view rather than that of researchers. This variation was used by Pelaccia and colleagues who examined how expert emergency physicians generated and evaluated diagnostic hypotheses as they worked.^7^

A common criticism of video-based research is that participants react to the camera and behave differently than they would if the camera were not present. A study was conducted to address the potential reactivity of cameras in medical interactions by filming interactions between 45 patients and 14 medical oncologists and then coding the film for camera-related behaviors.^8^ Results indicated that camera-related behaviors were infrequent (occurring 0.1% of the time) and most often occurred in very early stages of an interaction.

Diagnostic decision-making is a highly complex cognitive process that is susceptible to errors. There is inherent uncertainty in the diagnostic process, making it one of the most difficult tasks for the human mind.^9^ Unsurprisingly, emergency physicians are particularly vulnerable to making diagnostic errors because the emergency department (ED) is a fast-paced, dynamic setting where complex decision-making occurs under severe time, information, and resource constraints. By some estimates, there are about 7 million instances of error among the approximately 143 million visits to United States EDs every year, with half having a potential for patient harm.^10^ The purpose of this overall qualitative study was to describe how the diagnostic process evolves for emergency physicians in both pediatric and adult ED settings. We were interested in understanding how the diagnostic process unfolds for emergency physicians because those who work in EDs are particularly vulnerable to making diagnostic errors. We adapted Pelaccia’s method in which a physician wears a head-mounted camera to film their “own point-of-view” however, rather than using it to generate diagnostic hypotheses, we used their method to gain a better understanding of how the diagnostic process unfolds for emergency physicians

**Population Health Research Capsule**

What do we already know about this issue?

*ED physicians are vulnerable to making diagnostic errors. The pairing of video data and stimulated recall are well-suited to identify cognitive processes and learn from them*.

What was the research question?

*Can video with stimulated recall interviews describe how the diagnostic process evolves for ED physicians?*

What was the major finding of the study?

*This methodology uncovers the cognition of physicians during the diagnostic process to reveal how they think, as cognition is the heart of diagnosis*.

How does this improve population health?

*This methodology has the potential to reduce diagnostic error and thereby improve population health*.

When publishing research, word count limits in today’s journals restrict authors from providing the methodological detail needed to replicate or design future studies combining video and stimulated recall methods. As a result, conducting video-based stimulated recall interviews with participants after filming remains poorly described in the literature, limiting the ability of researchers to gain experience in this method, which is suited to explore cognitive processes and decision-making in medicine. In this paper, we provide details of the methods we used to first film physicians’ activities and subsequently conduct stimulated recall interviews with them. However, we did not formally test the proof of concept of combining video review with stimulated recall interviews in the real world with busy emergency physicians.

### Theoretical Model

We used the theory of distributed cognition to describe how information processing develops over time and occurs across various physicians, the technologies they use, and their social organization. Distributed cognition, a social cognitive theory, is gaining traction in healthcare because of the recognition that physicians work in social groups and the work is context dependent. There are three main principles of distributed cognition: the physical organization of work; the information flow; and the artifacts.^11^ All three principles must interact for distributed cognition to occur, underscoring the central premise of the theory that cognition is dispersed across people, places, and things.^12^

The physical organization of work refers to how work is configured and adapted to a specific context. It also includes things that can be seen, heard, and accessed by the people doing the work. The physical organization of work affects the second principle, information flow, which refers to the mechanism that allows a system to operate. Information flow is a bidirectional process that involves interactions between patient and/or family member and physician. Information flow is accomplished through communication using all mediums (eg, face to face, electronic, telephone) and types (eg, verbal, nonverbal).

Artifacts make up the final major principle in the theory of distributed cognition and refer to cognitive aids that support a physician’s ability to make a diagnosis. The most used artifact is the electronic health record (EHR), which maintains an account of a patient’s past history and present illness and serves as a repository for lab values and other diagnostic testing results. The EHR has many other uses such as providing templates for discharge instructions and patient education materials. Another artifact used in the ED is the white board,which displays patient names (or initials), physician room assignment, care team members who are working that shift, and status (ie, discharge, test results pending, admit). Other artifacts that support physicians’ cognition include paper and electronic reminders. The ED is unpredictable and uniquely dynamic compared to hospital or clinic settings^13^,^14^; thus, a theory that can describe how distinct attributes of the ED affect physicians’ thinking may help shed more light on the diagnostic process.

## METHODS

### Setting

The setting for our study was an academic medical and Level I trauma center in the midwestern United States that houses both an adult and pediatric ED.

### Recruitment

We used multiple strategies to recruit attending-level physicians as participants in the study. We first garnered the support of physician leadership of both EDs through brief one-on-one meetings. We developed a one-page flyer that was distributed via email to all attending emergency physicians. We attended a physician faculty meeting where we described the scope of the study and answered questions. Apprehension about being recorded during clinical practice is a known barrier to participation.^15^ To overcome this barrier, we framed participation as an opportunity to contribute to reducing diagnostic error and improving the quality of care.^15^ We also stressed that we were not critiquing clinical practice or competency. Once a physician agreed to participate and signed a consent form, a member of the research team scheduled a day and time for the video recording that aligned with a regularly scheduled clinical shift of the participant and the availability of two research team members. We conducted recruitment of 11 attending-level physicians (six in the adult ED and five in the pediatric ED; four were female, and seven male) on a rolling basis from February 2022–May 2023. We also notified other ED staff (eg, residents, medical students, physician assistants) about the study via e-mail and posted signs (while we were present and recording) for transparency.

Since nurses have a role as team members in the diagnostic process, we also met with ED nursing leadership to garner their support. Then we attended staff nurse huddles to describe the study and answer questions. We also distributed a one-page study information sheet in person and via email to to all nursing staff.

### Pre-Data Collection Procedures

Researchers spent a total of seven hours observing physicians in both EDs before any video recording occurred. The purpose of these preliminary observations was to understand the dynamic nature of ED operations and discover ways to minimize potential interruptions to physician workflow. Preparing for data collection also included testing the camera and microphone.

### Data Collection from Video

Two research team members collected video data across day and evening shifts. They followed a checklist of activities that was completed before the start of each session, during filming, and before leaving the unit. (See Supplemental Appendix A.) At the start of each session, research team members met briefly with the participant to identify patients who would be appropriate to approach. We included patients who were assigned to a participant and would be seen during their scheduled shift. We excluded patients deemed inappropriate by the participant (eg, critically ill or those with mental health complaints) and those who were awaiting disposition because they did not require further diagnostic decisions. We did not enter “special precautions” rooms (eg, patients under investigation for COVID-19 infection) to avoid the need to clean the equipment upon leaving the room. One member of the research team outfitted the participant with the head-mounted camera and a miniature, clip-on lavalier microphone to amplify the audio portion of recordings. We introduced participants to the equipment and allowed them to voice concerns or ask questions.

The ED is a noisy environment, and it was not always possible to hear the participant or their conversations. Initially, we planned on having two sources of audio capture (audio from camera and microphone) to provide adequate sound quality, but we had trouble capturing the voices of people talking with the participant, and sometimes ambient noise overpowered all voices. After the fifth recording session, we added a portable audio recorder that had two microphones. The center microphone captured dialogue while the side microphone captured environmental sound that was adjusted during postproduction to enhance overall sound quality.

### Roles of Research Team Members

Research team member roles and responsibilities are described in Table 1. Once recording began, research team member “A” conducted general observation to capture contextual features of the environment that otherwise would have been missed while the participant was wearing the camera; at the same time, research team member “B” took unstructured field notes. After two recording sessions we realized that rather than documenting observation field notes, research team member “B” was needed to enter the room ahead of the participant and inform patients of the study while research team member “A” stayed with the participant and took notes. Research team member “B” had several tasks: 1) deliver the prepared verbal script to each patient who was assigned to the participating physician and any family members/visitors and provide them with a one-page study overview; 2) secure patient approval to be filmed or give them the opportunity to decline the presence of a video camera in their room; and 3) note all requests to blur images of individuals caught incidentally on film to protect anonymity (eg, nurses who were also present in the room). Across recording sessions, 69 patients were briefed on the study, and 54 approved the use of recording equipment during the visit.

Without this division of labor, the participating physician was unable to work at their usual pace and had to slow down or wait for the research team to determine whether the next patient would agree to video recording. When participants needed to see patients who opted out or were excluded, all audio and visual recording was stopped, and research team member “A” helped the physician to remove equipment and then reattach it after the patient encounter was complete. During each session research team members also introduced themselves to other physicians, described the study, and answered any questions. They distributed one-page study information sheets and helped facilitate future recruitment and lessen any anxiety related to their presence.

The research team worked together to keep track of the cumulative recording time, aiming for two hours of audio and video data showing multiple participant-patient interactions for each participant. When recording was complete, research team member “A” removed equipment from the participating physician, thanked them for their time, and informed them that we would analyze data and schedule a follow-up interview to discuss what was captured on film. The research team was on site for 2.5–6 hours for each session to allow for preparations, equipment removal due to patient/family refusals, special precautions encounters, and resuscitation responses.

### Post Video Recording Procedures

Immediately after filming was done, research team member “A” returned to a designated research office and uploaded video and audio data files to a secure server. After confirming that all files had been securely uploaded, they were erased from the video and audio recorders. The camera and microphones were cleaned and recharged after each session. Before the research team reviewed video data, a research assistant with expertise in audio and video technologies combined the video and audio files, overlaying the audio data from the lavalier microphone and portable audio recorder onto video data, generating enhanced video data later used for analysis. Several other important tasks were also completed: using features to balance audio, they were able to increase volume, reduce background noise, and remove both low frequency static and high frequency buzzing sounds; they amplified the audio of individuals to whom the participant was speaking directly; and they placed a Gaussian blur on the faces of ED staff members who requested to be blurred from the video data.

### Data Collection from Stimulated Recall Interviews

We prepared interview guides for individual follow-up interviews with each participating physician, which were scheduled for one hour and conducted via video conferencing technology (ie, Zoom). From each recording session, we selected 3–4 short video clips related to each of the three concepts within our theoretical model (for a total of 9–12 clips) to display during interviews and prepared interview questions to accompany each video clip. For each exemplar clip, we developed a brief introduction to the clip, interview question, and probes designed to add context for the clip and elicit participants’ reflections on their decisions and diagnostic process. Supplemental Appendix B displays the number of video clips selected for each participant, along with the total and average length of each clip, arranged by questions according to the theory of distributed cognition. For example, during interview #3, we opted to explore further with the participant four clips where there was discussion of workflow, three clips that discussed how the EHR helped or hindered the diagnostic process, and six where there was discussion about interactions with others. Our goal was to keep the total time of video clips that we played during each interview to no more than 10 minutes to allow an in-depth conversation with each participant about their thoughts and feelings related to their diagnostic process.

Scheduling interviews with participants who were on service for a week or two at a time took longer than anticipated. After the first two interviews, we moved to a rapid analysis methodology to shorten the time from data collection to interview. Rapid analysis provides actionable, rigorous qualitative data in less time than traditional analysis.^16^ We developed a template based on the three major concepts of distributed cognition—our deductive framework—to help organize data (Table 2). Research team members independently reviewed video recordings to identify situations related to the diagnostic process or one of the three major concepts of our deductive framework. We met weekly to discuss our individual impressions of video footage and came to consensus on timestamps for clips that were the best examples of one of the three concepts of distributed cognition. Selected video clips were embedded into a PowerPoint presentation that was shown to the participating physcian during individual interviews.

A “dry run” was done prior to each interview to make sure that questions aligned with the selected video clip, verify video clip times (to ensure there was enough context on the clip for the participant to answer the question), and confirm that the wording was clear. Audio quality was further enhanced, if needed. Two research team members conducted each interview. One asked questions while the other advanced the slides and played video clips, as well as asking additional questions. All interviews were recorded, transcribed, and verified for accuracy.

An important consideration before undertaking a project such as ours is cost. The head-mounted camera we used (including mounting brackets) cost $188 and included software to download videos onto a laptop computer. We did not purchase a project-specific laptop, but these have a wide range of prices ($550 – $1,800). The lavalier microphone cost $29 (range $11–$70) and the Zoom microphone cost $200 ($100–$280). We did not purchase video editing software, which ranges in price between $40–$264. As technology advances, costs for audio and video equipment have been declining, making it feasible to conduct projects without great expense.

## DISCUSSION

We report a methodology that we used to describe how the diagnostic process evolves for emergency physicians in both pediatric and adult ED settings. There are several advantages to our approach that warrant discussion. First, the type of method dictates the kind of data that will be generated because each method captures a specific type of data.^19^ The use of video provided us with details of the ED environment that could not be captured by observation or interview alone.^20^ Observation is limited to those select activities that an observer can notice at any one time, and interviews provide a retrospective view of the opinions of the interviewees rather than a window into the here-and-now, as is possible with video. Given the busy, chaotic, and interruption-driven nature of EDs, the unique perspective provided by video allowed us to understand the context of the physician’s encounter, while stimulated recall interviews revealed its effect on their thought processes.

A second advantage to video is the ability to rewind and review what was on the film. To improve efficiency and facilitate throughput in a fast-paced ED, physicians usually multitask and engage in several activities at the same time. Without the ability to rewind and review a video, important and subtle nuances of the diagnostic process as manifested by concurrent activities could be lost. Up to 80% of communication may be nonverbal,^21^ and since communication plays such a large role in the diagnostic process,^22^ video captures these nonverbal communication behaviors (eg, body language, eye gaze) with high fidelity and a greater level of detail than other observational methods. Finally, by having the physician wear the camera, what was captured indirectly was the physician’s thought processes as they looked at a person, the computer screen, or artifact such as a piece of paper. In subsequent stimulated recall interviews, we were able to confirm or refute what the physician remembered about their own thoughts and feelings in the moment.

Stimulated recall interviews provided another type of data. Prompts provided by video clips during interviews allowed participants to describe thoughts and feelings as they organized their work or communicated with others. Associated interview probes gave participants the opportunity to expound on their thinking and provide the rationale for their activities, thereby extending the information gained.

One of the earliest uses of head-mounted video capture followed by stimulated recall interviews in healthcare was in the field of occupational therapy to study the therapist’s clinical reasoning while working with a patient.^23^ More recently, video-stimulated recall has been used to understand differences in perspectives between the healthcare team and family members during family-centered rounds at a pediatric hospital,^24^ to examine the primary care physician-patient consultation process,^25^ and to analyze interactions between physicians and patients with osteoarthritis.^14^ The wide variety of applications of this method suggests that reflection while watching video and deliberation during recall interviews have value beyond either method in isolation.

The use of head-mounted video cameras in healthcare applications without subsequent stimulated recall interviews is growing, although the video is used for different purposes post-filming. More recently, head-mounted video cameras have been used to teach surgical residents in the operating room,^26^,^27^ to train learners on simulated intubation procedures,^28^ and to train pulmonary critical care medicine fellows in performing thoracentesis on actual patients.^29^ In these cases, post-video film provided objective evaluation of skill development.

To the best of our knowledge, there has been very limited use of head-mounted cameras and post-filming stimulated recall interviews to understand how emergency physicians think or develop diagnoses. Our study and the study by Pelaccia and colleagues are the only ones to date that have sought to uncover cognitive processes related to diagnosis; however, our methods differed in significant ways. In the study by Pelaccia, each filmed encounter ranged from 8.5–20.5 minutes in duration, which is considerably less than the two hours of film generated for each of the 11 physicians in our study. This speaks to the difference in objectives between the two studies. Pelaccia found that hypothesis generation was primarily an intuitive process and that most physicians had formulated a hypothesis even before initially seeing the patient.^7^ Because they were interested in capturing each physician’s initial hypothesis, they sought to minimize the effects of recall bias by conducting interviews within an hour or two after recording the encounter. In contrast, on average 60 days elapsed between filming an encounter and the recall interview in our study.

Because diagnostic error is a multifaceted phenomenon, many approaches will be needed to reduce it, particularly in the chaotic ED setting. Technological advances,^30^ improving communication,^31^ and listening better to patients and family members^32^,^33^ are some of the more common approaches being used and these employ a host of methods. The strength of the methodology we describe is in uncovering the cognition of physicians during the diagnostic process to reveal how they think because cognition is at the heart of diagnosis.

## LIMITATIONS

The use of video coupled with stimulated recall interviews to capture visible practice and invisible cognitive processes is a promising methodology for studying complex processes such as diagnosis. However, there are several limitations associated with this methodology. As with all qualitative methods, the aim of video-based research is to develop an in-depth understanding of some phenomena rather than to achieve generalizability; thus, its scalability and wider application are limited. The camera, even one as small and unobtrusive as the one we used, becomes part of the research and introduces the possibility of a Hawthorne effect, although such effects do appear to be small.^6^,^17^,^18^

On average, 60 days elapsed between filming and recall interview for many participants, reducing the accuracy and trustworthiness of responses.^3^ Participants made comments, such as “I don’t remember this case” or “I remember this one,” which did not appear to be associated with the time delay between when the video was recorded and the interview. Finally, it is not clear whether in reviewing the video clips the participating physicians were recalling their thoughts and feelings from the time the video was taken or were reacting to what they were viewing in the present.^3^ Thus, narratives produced from review may not represent the conscious or unconscious cognitions taking place at the time of the video recorded episode.^3^

## CONCLUSION

Video recording a physician’s clinical practice in the emergency department as it occurs, followed by video-stimulated recall interviews with them, has the potential to advance our understanding of the diagnostic process. From the physician’s perspective, becoming aware of how one thinks allows what was hidden to become visible and, once visible, more amenable to reflection and change, to bring us closer to reducing diagnostic error.

## Supplementary Information





## Figures and Tables

**Table 1:** Team structure and team responsibilities in a video-based study describing how the diagnostic process evolves for emergency physicians.

Research team member	Qualifications	Responsibilities
Project manager	Master’s degree in Public Health. Over five years’ experience as a project manager	Recruit participants, secure informed consent, schedule recording sessions, coordinate activities, edit audio/video if needed
Clinical research coordinator (CRC)	PhD candidate in anthropology with training in ethnography	Maintain equipment (camera and microphone), complete the checklist, lead research activities on site, outfit the participant with recording equipment, work with the participant and research team members to identify patients and families who needed to be informed and approve of or opt out of recording, enter patient rooms to gain approval or opt-out when the RA was unavailable, shadow the participant while wearing the camera, take field notes, upload and organize data files, communicate the need for anonymity measures (blurring)
Research team member “A”	Undergraduate or graduate student with nursing or pre-medicine major, or full-time ED research coordinator	Help prepare equipment, post notices of recording on site, work with the CRC and participant to identify patients and families who needed to be informed and approve of or opt out of recording
Research team member “B”	Undergraduate or graduate student with nursing or pre-medicine major, or full-time ED research coordinator	Enter the patient’s room ahead of time to secure approval, note requests to blur images of those caught incidentally on camera, assist with cleaning and checking equipment and uploading data or recording field notes, if needed
Research assistant (RA)	Specific audio and video technology expertise.	Combine video and audio files, improve audio quality, place Gaussian blur on faces of those who requested it
Co-investigator	PhD-prepared professor of nursing with experience in video-based studies	Replace either the CRC or RA, as needed

**Table 2:** “Rapid” video analysis template used in a video-based qualitative study describing how the diagnostic process evolves for emergency physicians.

Examples of Workflow			
Video #	Start time	Stop Time	Why am I including this clip?
			
			
			
			
**Examples of EHR and Artifacts**			
Video #	Start time	Stop Time	Why am I including this clip?
			
			
			
			
**Examples of Interactions with Others**			
Video #	Start time	Stop Time	Why am I including this clip?
			
			
			
			

Instructions for Analysts: While watching a video think about which of three categories might best fit what you are noticing: 1) How does the physician arrange or organize their workflow, and what are the implications of that workflow pattern for diagnosis (includes interruptions)? 2) How do the EHR and other artifacts (such as paper notes, white board) help or hinder the diagnostic process? 3) How do interactions with others affect the diagnostic process (can be with other clinicians, nurses, allied health personnel, or with patients/family members)? Every time you notice something that fits into one of the three categories, please also note the start and stop time stamps for that activity and document it in the appropriate box below. Add as many rows as you would like, and please remember that we will also have to jointly choose the top three clips as bests examples of each category.

*EHR*, electronic health record.
