# Supplementary material for: Using “Own-Point-of-View” Video and Stimulated Recall to Reveal Physician Thought Processes: A Methodology Paper
Source: West J Emerg Med. 2026 Jul 16;27(4):1073–81. doi: 10.5811/westjem.54032 (PMC13436663; doi:10.5811/westjem.54032)
Supplement: Supplementary file 1 [file wjem-27-1073-s001.docx]

Supplemental Appendix A: Methodological Procedures

- Principal investigator and co-investigator present to nursing and physician groups
  - Explain study and who will/will not be recorded, who will/will not be consented
    - Ability to opt-out (and what that means for recordings)
- Based on above feedback, can draft observation schedule based on those available to observe & can begin pre-consenting process and limit need to consent day-of
- Starting 1-4 weeks prior to observation -
  - Project manager will consent attending physicians using SignNow – consenting physician subject prior to observation day
- Four to five days before –
  - send “blast” email to all nursing staff of the adult and pediatric EDs where video recording will occur.
  - Include attachment (single page, bulleted study protocol “One pager for nurses”).
  - Post flyers on the unit. Find out from leadership where the best place is to post flyers for both nurses and physicians.
- One to two days before –
  - Send a second email to all nursing staff of the adult and pediatric EDs where video recording will occur.
  - Contact charge nurse to alert them as well to the upcoming video recording session.
- Day of video recording –
  - Post notice at nursing station about time and duration of video recording.
  - Get advice from staff on which patient rooms not to enter (e.g., confused and frightened by unknown groups of people, combative).
  - Alert all patients/families already in the ED who are on the participant’s panel of patients for that shift, reading verbal script to them. Patients/family can opt out.
  - Obtain permission from nurses with whom participating physicians have a conversation about a patient AND whose images are captured on the camera using prepared verbal script for general observation and offer one page study information sheets. If non-consenting nurse images are captured on film, they will be deleted from the video, or blurred.
  - Nurses may be asked to participate in short interviews after filming to provide their perspective on what occurred.
  - Obtain permission from physician assistants (PAs), residents with whom participating physicians have a conversation about a patient AND whose images are captured on the camera using prepared verbal script for general observation and offer one page study information sheets. If non-consenting PA/resident images are captured on film, they will be deleted from the video, or blurred.
  - Obtain permission from other patient care team members who are not subjects of the study (e.g., nurses, pharmacists, care coordinators).
- Before leaving for the day –
  - Brief interview with any nurses who participated in conversation with participating physicians.
  - Schedule participating physician for follow-up interview.
