# Supplementary material for: Using “Own-Point-of-View” Video and Stimulated Recall to Reveal Physician Thought Processes: A Methodology Paper
Source: West J Emerg Med. 2026 Jul 16;27(4):1073–81. doi: 10.5811/westjem.54032 (PMC13436663; doi:10.5811/westjem.54032)
Supplement: Supplementary file 2 [file wjem-27-1073-s002.docx]

Supplemental Appendix B: Number of video clips selected for each participant interview

|  | **How do you arrange your workflow, and what are the implications of your workflow for diagnosis?** | | | **How does the EHR help or hinder  your ability to make a diagnosis?** | | | **How do interactions with others  affect diagnostic decision making?** | | |
| --- | --- | --- | --- | --- | --- | --- | --- | --- | --- |
|  | Video Clips | Total Clip Length (mm:ss) | Average Clip Length (mm:ss) | Video Clips | Total Clip Length (mm:ss) | Average Clip Length (mm:ss) | Video Clips | Total Clip Length (mm:ss) | Average Clip Length (mm:ss) |
| Interview 1 | 5 | 03:06.4 | 00:37.3 | 5 | 03:24.0 | 00:40.8 | 6 | 04:20.0 | 00:43.3 |
| Interview 2 | 3 | 00:36.3 | 00:12.1 | 3 | 00:27.5 | 00:09.2 | 8 | 03:39.0 | 00:27.4 |
| Interview 3 | 4 | 01:50.6 | 00:27.6 | 3 | 00:53.2 | 00:17.7 | 6 | 04:42.5 | 01:20.7 |
| Interview 4 | 4 | 02:37.6 | 00:39.4 | 4 | 02:24.8 | 00:38.6 | 4 | 03:41.8 | 00:55.5 |
| Interview 5 | 0 | 00:00.0 | 00:00.0 | 4 | 02:24.8 | 00:36.2 | 8 | 05:49.9 | 00:43.7 |
| Interview 6 | 5 | 01:42.5 | 00:20.5 | 4 | 01:15.4 | 00:18.9 | 3 | 02:24.5 | 00:48.2 |
| Interview 7 | 3 | 01:22.8 | 00:27.6 | 3 | 01:16.4 | 00:25.5 | 4 | 02:45.0 | 00:41.2 |
| Interview 8 | 4 | 02:38.2 | 00:39.6 | 3 | 01:31.7 | 00:30.6 | 4 | 02:59.7 | 00:44.9 |
| Interview 9 | 3 | 01:46.0 | 00:35.3 | 4 | 01:54.1 | 00:28.5 | 5 | 03:28.8 | 00:41.8 |
| Interview 10 | 3 | 04:10.3 | 01:23.4 | 3 | 03:00.8 | 01:00.3 | 3 | 01:53.1 | 00:37.7 |
| Interview 11 | 1 | 01:09.4 | 01:09.4 | 2 | 01:11.4 | 00:35.7 | 4 | 02:22.8 | 00:35.7 |
| AVERAGE | 3.18 | 01:54.6 | 00:35.7 | 3.45 | 01:47.6 | 00:31.1 | 5 | 03:27.9 | 00:45.5 |
|  |  |  |  |  |  |  |  |  |  |
| OVERALL |  |  |  |  |  |  |  |  |  |
| Avg number clips per interview question | | | | 3.88 |  |  |  |  |  |
| Avg number clips per interview | | |  | 11.64 |  |  |  |  |  |
| Avg video per interview question | | |  | 02:23.4 |  |  |  |  |  |
| Avg total video per interview | | |  | 07:10.1 |  |  |  |  |  |
| Avg clip length | |  |  | 00:37.4 |  |  |  |  |  |
| Range number clips per question per interview | | | | 0 | 8 |  |  |  |  |
